# Editorial: Crop response to density: Optimization of resource use to promote sustainability

**DOI:** 10.3389/fpls.2022.969332

**Published:** 2022-08-02

**Authors:** Ioannis Tokatlidis, Yashvir Chauhan, Yared Assefa

**Affiliations:** ^1^Department of Molecular Biology and Genetics, Democritus University of Thrace, Alexandroupoli, Greece; ^2^Department of Agriculture and Fisheries, The University of Queensland, Brisbane, QLD, Australia; ^3^Department of Agronomy, Kansas State University, Manhattan, KS, United States

**Keywords:** crop resilience, competition, grain yield efficiency, low-input agriculture, yield gap

This issue centered around plant population density and related topics; those were stem lodging and kernel abortion, decline and variability in solar radiation, leaf area index and the amount of intercepted photosynthetically active radiation, variability in optimum density and other topics for maize and other crops (sweet corn, wheat, grain sorghum, and barley).

To prevent a general food crisis, one of the major challenges facing agricultural research today is bridging a considerable yield gap. The yield gap is due to inefficient use of natural resources, resulting in harvested yield that lags behind the attainable yield. Among several factors contributing to the yield gap, the inability of individual plants to sufficiently capture inputs is a radical source. Low “plant yield efficiency” drastically affects the required number of plants per area, i.e., the plant population density (crop population); since water is the most crucial input, the phenomenon is more pronounced in rainfed crops. There is a consensus that under stressful conditions (i.e., drought), optimal resource use is accomplished only in low crop populations. On the other hand, due to the inability of individual plants to respond to additional inputs, modern varieties may reach the attainable yield of favorable environments at high crop populations. As conditions of growing seasons are difficult to predict during the growing period, the established plant population density may deviate from the one suitable for the season, and farmers may sustain a yield penalty. Crop modeling is helping to overcome this limitation but incorporating greater resource use plasticity could be a way forward.

The maize (*Zea mays* L.) collapse events of 2012 in Iowa and 2018 in Germany indicate that crop adaptation to spacing (low populations) is imperative to avoid crop failure in dry seasons without compromising the attainable grain yield during favorable seasons. As one of the topic editors previously emphasized (Tokatlidis, 2013, 2014, 2017), substantial benefits arise from crop adaptation to spacing: mitigation of the acquired plant-to-plant variability to optimize further the resource use; better compensation in both the common situation of missing plants and when multiline or open pollinated varieties are preferred to counteract unpredictable acute stresses (in both cases, individual plants would be able to utilize the input share of missing neighbors); adaptation to crop spacing would also expand the optimal planting date; adopting low-input cropping where necessary would prevent soil degradation and protect natural resources and the environment.

The objective of our collection was to attract articles from the fields of breeding, agronomy, physiology, soil science, molecular and genomic approaches related to the crop by population interaction, and in particular: crop response to population across varying environments; plant physiological response to crop population; interplant competition within a crop and breeding to mitigate the consequent intra-crop variation; soil water and soil biological and physicochemical properties that may relate to plant response to crop population; yield components related to crop population; environmental indicators for the optimal crop population; molecular and genomics that relate to crop population.

In this collection, most of the accepted submissions (7 out of 11) concerned maize, a crop whose average crop population has increased over the years across the globe (Assefa et al., 2018). A significant grain yield increase was associated with increased crop population, mainly in high- to medium-yielding environments (Assefa et al., 2016). The main questions addressed are the consequences of increased crop population and possible solutions. One of the articles (Shah et al.) deliberates that stem lodging and kernel abortion are major constraints in maize grain yield production as the crop population increases. Therefore, it is crucial to overcome stem lodging and kernel abortion, and Shah et al. review address that concern. The other shortcoming with increasing the crop population is a decline and variability in solar radiation that reaches each plant due to shading, affecting the crop productivity drastically; the issue is addressed by Yang et al. Zhang et al. reported a significant increase in leaf area index and the amount of intercepted photosynthetically active radiation with increased crop population. However, increased plant population reduced photosynthetic capacity, stomatal conductance, leaf chlorophyll content, and other responses, which are vital for crop productivity and yield stability. Capitalizing on the point that a high crop population aggravates competition among plants and harms plant growth and productivity, Liu et al. (2022) presented results proposing that nitrogen application and chemical control may improve plant growth and increase grain yield in a high crop population.

Crop population is a function of row spacing and plant spacing. An optimal combination could result in the same crop population but better resource use efficiency and higher productivity achieved through better planting configuration. Row- and plant-spacing should also be considered in conjunction with different soil and crop management. Indeed, Haarhoff and Swanepoel address the issue and accomplish increased light interception in a no-till semi-arid environment. Capitalizing on the same subject, Winans et al., beyond row spacing, explored other agronomic inputs such as P-S-Zn fertility, K-B fertility, N fertility, and foliar protection that could alleviate density-induced stress in a high crop population. In light of this, one might look for the key to identifying maize hybrids that tolerate high crop populations. Larrosa and Borrás answer that there is a relationship between density tolerance and radiation reduction around flowering.

Other publications of the collection discuss the issue of crop population in sweet corn (Dhaliwal et al.), wheat (Jaenisch et al.), grain sorghum (Zhou et al.), and barley (Tsivelikas et al.). Similar to points raised in maize, these papers also discuss planting density trends, yield component compensation, yield and quality response, and possible hybrid selection tools for high crop populations. From the breeding perspective, Tsivelikas et al. deal with interplant distance as a factor affecting the efficiency of single-plant selection, suggesting the absolute absence of inter-genotypic competition. We believe the information compiled delivers important Research Topics regarding crop response to planting density and raises new breeding challenges. However, we expected greater contribution from a breeding point of view, particularly in adapting crops to lower densities, stabilizing optimum density and creating varieties capable of effective resource use in variable environments, and reducing the yield gap (Tokatlidis, 2017; Fischer, 2020). The collection did not intensively cover historical trends in maize plant density reliance and plant density relations, which we assumed are covered in prior publications. We encourage readers to look into previous publications of the editors related to these areas as further reading (e.g., Tokatlidis and Koutroubas, 2004; Tokatlidis et al., 2011; Tokatlidis, 2013, 2014; Assefa et al., 2016, 2018; Solomon et al., 2017; Mylonas et al., 2020). Tokatlidis (2017) argues that to reach crop adaptation to lower populations and resilience, breeding for density-independent varieties *via* improved plant yield efficiency is a viable option and imperative to bridge the yield gap. Also, the effects of plant density on root systems and its consequence on the efficiency of the use of below ground resources, including their influence on soil microflora, were not extensively covered in this collection, and we suggest further issues to cover these gaps.

## Author contributions

All authors listed have made a substantial, direct, and intellectual contribution to the work and approved it for publication.

## Conflict of interest

The authors declare that the research was conducted in the absence of any commercial or financial relationships that could be construed as a potential conflict of interest.

## Publisher's note

All claims expressed in this article are solely those of the authors and do not necessarily represent those of their affiliated organizations, or those of the publisher, the editors and the reviewers. Any product that may be evaluated in this article, or claim that may be made by its manufacturer, is not guaranteed or endorsed by the publisher.

## References

[B1] Assefa Y. Carter P. Hinds M. Bhalla G. Schon R. Jeschke M.. (2018). Analysis of long term study indicates both agronomic optimal plant density and increase maize yield per plant contributed to yield gain. Sci. Rep. 8, 4937. 10.1038/s41598-018-23362-x 29563534 PMC5862987

[B2] Assefa Y. Vara Prasad P. V. Carter P. Hinds M. Bhalla G. Schon R.. (2016). Yield responses to planting density for us modern corn hybrids: a synthesis-analysis. Crop Sci. 56, 2802–2817. 10.2135/cropsci2016.04.0215

[B3] Fischer R. A. (2020). Breeding wheat for increased potential yield: contrasting ideas from Donald and Fasoulas, and the case for early generation selection under nil competition. Field Crops Res. 252, 107782. 10.1016/j.fcr.2020.107782

[B4] Liu X. Zhang L. Yu Y. Qian C. Li C. Wei S.. (2022). Nitrogen and chemical control management improve yield and quality in high-density planting of maize by promoting root-bleeding sap and nutrient absorption. Front Plant Sci. 13, 754232. 10.3389/fpls.2022.754232 35812983 PMC9260249

[B5] Mylonas I. Sinapidou E. Remountakis E. Sistanis I. Pankou C. Ninou E.. (2020). Improved plant yield efficiency alleviates the erratic optimum density in maize. Agron. J. 112, 1690–1701. 10.1002/agj2.20187

[B6] Solomon K. F. Chauhan Y. Zeppa A. (2017). Risks of yield loss due to variation in optimum density for different maize genotypes under variable environmental conditions. J. Agron. Crop Sci. 203, 519–527. 10.1111/jac.12213

[B7] Tokatlidis I. S. (2013). Adapting maize crop to climate change. Agron. Sustain. Dev. 33, 63–79. 10.1007/s13593-012-0108-7

[B8] Tokatlidis I. S. (2014). Addressing the yield by density interaction is a prerequisite to bridge the yield gap of rainfed wheat. Ann. Appl. Biol. 165, 27–42. 10.1111/aab.12121

[B9] Tokatlidis I. S. (2017). Crop adaptation to density to optimise grain yield: breeding implications. Euphytica 213, 92. 10.1007/s10681-017-1874-8

[B10] Tokatlidis I. S. Has V. Melidis V. Has I. Mylonas I. Evgenidis G.. (2011). Maize hybrids less dependent on high plant densities improve resource use efficiency in rainfed and irrigated conditions. Field Crops Res. 120, 345–351. 10.1016/j.fcr.2010.11.006

[B11] Tokatlidis I. S. Koutroubas S. D. (2004). A review study of the maize hybrids' dependence on high plant populations and its implications on crop yield stability. Field Crops Res. 88, 103–114. 10.1016/j.fcr.2003.11.013

